# Unique Microstructural Changes in the Brain Associated with Urological Chronic Pelvic Pain Syndrome (UCPPS) Revealed by Diffusion Tensor MRI, Super-Resolution Track Density Imaging, and Statistical Parameter Mapping: A MAPP Network Neuroimaging Study

**DOI:** 10.1371/journal.pone.0140250

**Published:** 2015-10-13

**Authors:** Davis Woodworth, Emeran Mayer, Kevin Leu, Cody Ashe-McNalley, Bruce D. Naliboff, Jennifer S. Labus, Kirsten Tillisch, Jason J. Kutch, Melissa A. Farmer, A. Vania Apkarian, Kevin A. Johnson, Sean C. Mackey, Timothy J. Ness, J. Richard Landis, Georg Deutsch, Richard E. Harris, Daniel J. Clauw, Chris Mullins, Benjamin M. Ellingson, MAPP Research Network

**Affiliations:** 1 Department of Radiological Science, David Geffen School of Medicine, University of California Los Angeles, Los Angeles, California, United States of America; 2 Department of Biomedical Physics, David Geffen School of Medicine, University of California Los Angeles, Los Angeles, California, United States of America; 3 Oppenheimer Center for the Neurobiology of Stress, and PAIN, David Geffen School of Medicine, University of California Los Angeles, Los Angeles, California, United States of America; 4 Department of Digestive Diseases and Gastroenterology, David Geffen School of Medicine, University of California Los Angeles, Los Angeles, California, United States of America; 5 Department of Psychiatry and Biobehavioral Sciences, David Geffen School of Medicine, University of California Los Angeles, Los Angeles, California, United States of America; 6 Department of Bioengineering, David Geffen School of Medicine, University of California Los Angeles, Los Angeles, California, United States of America; 7 Department of Physiology, Northwestern University, Chicago, Illinois, United States of America; 8 Department of Neurology, Stanford University, Palo Alto, California, United States of America; 9 Department of Anesthesiology, University of Alabama, Birmingham, Alabama, United States of America; 10 Department of Biostatistics and Epidemiology, Perelman School of Medicine at the University of Pennsylvania, Philadelphia, Pennsylvania, United States of America; 11 Department of Radiology, University of Alabama, Birmingham, Alabama, United States of America; 12 Department of Anestesiology, University of Michigan, Ann Arbor, Michigan, United States of America; 13 Division of Kidney, Urologic, and Hematologic Diseases; National Institute of Diabetes and Digestive and Kidney Diseases; National Institutes of Health, Bethesda, Maryland, United States of America; 14 Division of Biokinesiology and Physical Therapy, University of Southern California, Los Angeles, California, United States of America; Chinese Academy of Sciences, CHINA

## Abstract

Studies have suggested chronic pain syndromes are associated with neural reorganization in specific regions associated with perception, processing, and integration of pain. Urological chronic pelvic pain syndrome (UCPPS) represents a collection of pain syndromes characterized by pelvic pain, namely Chronic Prostatitis/Chronic Pelvic Pain Syndrome (CP/CPPS) and Interstitial Cystitis/Painful Bladder Syndrome (IC/PBS), that are both poorly understood in their pathophysiology, and treated ineffectively. We hypothesized patients with UCPPS may have microstructural differences in the brain compared with healthy control subjects (HCs), as well as patients with irritable bowel syndrome (IBS), a common gastrointestinal pain disorder. In the current study we performed population-based voxel-wise DTI and super-resolution track density imaging (TDI) in a large, two-center sample of phenotyped patients from the multicenter cohort with UCPPS (*N* = *45*), IBS (*N = 39*), and HCs (*N = 56*) as part of the MAPP Research Network. Compared with HCs, UCPPS patients had lower fractional anisotropy (FA), lower generalized anisotropy (GA), lower track density, and higher mean diffusivity (MD) in brain regions commonly associated with perception and integration of pain information. Results also showed significant differences in specific anatomical regions in UCPPS patients when compared with IBS patients, consistent with microstructural alterations specific to UCPPS. While IBS patients showed clear sex related differences in FA, MD, GA, and track density consistent with previous reports, few such differences were observed in UCPPS patients. Heat maps illustrating the correlation between specific regions of interest and various pain and urinary symptom scores showed clustering of significant associations along the cortico-basal ganglia-thalamic-cortical loop associated with pain integration, modulation, and perception. Together, results suggest patients with UCPPS have extensive microstructural differences within the brain, many specific to syndrome UCPPS versus IBS, that appear to be localized to regions associated with perception and integration of sensory information and pain modulation, and seem to be a consequence of longstanding pain.

## Introduction

Urological Chronic Pelvic Pain Syndrome (UCPPS) represents a group of pain syndromes relating to male and female pelvic pain that are poorly understood and for which treatment is largely unsatisfactory [1–3]. Since 2007 the National Institute of Diabetes and Digestive and Kidney Diseases (NIDDK) has combined Chronic Prostatitis/Chronic Pelvic Pain Syndrome (CP/CPPS) and Interstitial Cystitis/Painful Bladder Syndrome (IC/PBS) into a single classification of UCPPS [4]. The complex and largely unknown etiology, as well as the varied symptom presentation in UCPPS, presents tremendous difficulties in both diagnosis and classification [5–7]. This complexity inherent in UCPPS suggests that some potential combination of peripheral and central nervous system biomarkers may be the best approach to diagnosis, and may lead to a better understanding of the condition itself. For example, a functional magnetic resonance imaging (fMRI) brain study has recently shown functional differences between CP/CPPS patients and healthy controls (HCs) that seem unique to this specific pathophysiology [8]. Other non-invasive neuroimaging studies have also suggested structural and functional differences may exist between various pain conditions and HCs, including Irritable Bowel Syndrome (IBS) [9, 10], chronic back pain [11–13], chronic pancreatitis [14], and chronic complex regional pain syndrome (CRPS) [15]. The Multidisciplinary Approach to the Study of Chronic Pelvic Pain (MAPP) Research Network (**S1 Fig**) was developed to better understand the underlying pathophysiology of UCPPS and inform clinical management of patients [1, 16]. One important goal of the MAPP Network is to identify potential biological markers for UCPPS.

Diffusion weighted imaging (DWI) is a noninvasive magnetic resonance imaging (MRI) technique that can be used to measure the random motion of extracellular water molecules. Diffusion tensor imaging (DTI) is a specific DWI technique that fits a mathematical tensor to diffusion MR measurements obtained in various orientations, allowing sub-voxel microstructural information relating to both the magnitude and direction of water diffusivity to be quantified for every image voxel. DTI indices such as fractional anisotropy (FA), a metric relating to the relative degree of directionally-specific restricted diffusion, and mean diffusivity (MD), a metric relating to the average magnitude of water diffusion in all directions, have been used to identify differences in the brains of HCs and patients with various chronic pain conditions. For example, FA was found to be altered in cortico-thalamic circuits in male and female IBS patients as compared to HCs [10], while differences in MD were also observed. In a separate study, Chen *et al*. found higher FA in predefined regions of the fornix and external capsule for IBS patients compared to HCs [9]. Similarly, a recent DTI study of back pain showed that the average FA from a number of pooled regions could predict transition from acute to chronic back pain [17]. Other chronic pain conditions have also been studied using DTI, including chronic CRPS [15], chronic pancreatitis [14], and fibromyalgia [18], suggesting DTI and other diffusion MRI techniques may be adequately sensitive to subtle neural reorganization, which may accompany chronic pain conditions.

While second-order diffusion tensor metrics such as FA and MD have become a common measure used in clinical and research studies, there are limitation to using these measures: in voxels with more complex fiber organization, such as regions of crossing fibers, they are insufficient to describe the underlying microstructure. One potential way of overcoming these limitations is to use a higher-order tensor to more accurately model the underlying anatomy. This is the case for the 4^th^ order covariance tensor [19], which has shown promise for quantifying microstructural information and for tractography [20]. We proposed to use generalized anisotropy (GA), a measure of diffusion anisotropy based off the 4^th^ order tensor, in conjunction with FA and MD to evaluate the microstructural differences present in the brains of UCPPS subjects.

Super-resolution track density imaging (TDI) is a diffusion imaging technique first developed by Calamante *et al*. [21] that involves creation of high resolution white matter images by weighting each image voxel with information relating to the number of fiber tracts passing through each voxel after performing probabilistic tractography. TDI has been used to probe high-resolution white matter and compare it to normal brain histology in several animal models [22–24], used to visualize thalamic structures at high-resolution in comparison to high-field, high-resolution anatomical MRI [25], and was studied as a potential marker for tumor aggressivity in glioblastoma [26].

The characterization of brain signatures in UCPPS is important as it can help to identify neuroanatomical correlates of chronic pain, potential biomarkers as biological endpoints for clinical trials, and potential targets for therapies. The purpose of the current study was therefore to identify structural brain signatures, which are unique to patients with UCPPS. Specifically, we aimed to: 1) Determine whether there are spatially-specific differences in TDI, FA, GA and MD measurements in the brains of patients with UCPPS compared with HCs (HC) and with a positive control group of IBS subjects. 2) Identify possible sex related differences in DTI and TDI measurements within patients with UCPPS and contrast them with sex differences that may be present in HC or IBS patients. 3) Identify possible correlations between DTI or TDI measurements in the brain of UCPPS patients and symptom severity information.

## Materials and Methods

### Patient Population

All subjects provided informed written consent to participate in the current study. All consenting procedures and protocols were approved by the institutional review board at each of the participating sites, which included the University of California, Los Angeles (UCLA), Northwestern University (NWU), University of Michigan (UM), Stanford University (SU), and University of Alabama, Birmingham (UAB). Only subjects from UCLA and NWU with DTI data were used in the current study for reasons related to the availability and consistency of diffusion MR data, as described in a subsequent section. All subjects included in the study (**Table 1**) were right handed, with 56 HCs (30 male, 26 female, average age = 38 ± 13 years), 39 positive controls with IBS (16 male, 23 female, age = 35 ± 12 years), and 45 UCPPS subjects (26 male, 19 female, age = 40 ± 14 years, average symptom duration = 9 ± 11 years). Inclusion criteria for UCPPS participants consisted of [1, 16]: 1) a diagnosis of IC/BPS or CP/CPPS, with urologic symptoms present a majority of the time during any 3 of the past 6 months (CP/CPPS) or the most recent 3 months (IC/BPS); 2) at least 18 years old; 3) reporting a non-zero score for bladder/prostate and/or pelvic region pain, pressure or discomfort during the past 2 weeks; and 4) consented to provide a blood or cheek swab sample to test DNA for genes related to the main study goals. Exclusion criteria for UCPPS consisted of the following: symptomatic urethral stricture, on-going neurological conditions affecting the bladder or bowel, active autoimmune or infectious disorders, history of cystitis caused by tuberculosis or radiation or chemotherapies, history of non-dermatologic cancer, current major psychiatric disorders, or severe cardiac, pulmonary, renal, or hepatic disease. In addition, males diagnosed with unilateral orchalgia without pelvic symptoms, and males with a history of microwave thermotherapy, trans-urethral or needle ablation or other specified prostate procedures were excluded. Healthy control participants were excluded if they reported any pain in the pelvic or bladder region or chronic pain in more than one non-urologic body region. Like HCs, positive controls needed to be free of pain in the pelvic region, but also needed to qualify on the CMSI as having one of the targeted co-morbid conditions. For more specific inclusion and exclusion criteria, please see Landis *et al*. [16].

**Table 1:** Patient Characteristics. Values reported as mean ± standard deviation.

	HC (n = 56)	IBS (n = 39)	UCPPS (n = 45)	Group effect (p-value)	UCPPS vs. IBS (p-value)
Age	37.9 ± 12.9	35.3 ± 12.4	39.6 ± 14.3	0.31	-
Sex	30 M, 26 F	16 M, 23 F	26 M, 19 F	-	-
Body mass index	24.5 ± 4.4	24.2 ± 5.5	25.5 ± 5.4	0.42	-
UCPPS Symptom duration (years)	N/A	N/A	8.8 ± 11.4		
Hospital Anxiety and Depression Scale (HADS)					
Anxiety	3.3 ± 3.2	5.8 ± 5.2	8.0 ± 3.8	< 0.0001	0.03
Depression	2.3 ± 2.5	3.6 ± 2.9	5.8 ± 3.7	< 0.0001	0.003
Genitourinary Pain Index (GUPI)					
Pain Domain	0.2 ± 0.9	3.1 ± 3.6	11.8 ± 4.2	< 0.0001	< 0.0001
Urinary Domain	0.8 ± 1.4	1.7 ± 2.0	4.8 ± 2.9	< 0.0001	< 0.0001
Quality of Life Domain	0.6 ± 1.5	3.6 ± 3.4	7.4 ± 2.8	< 0.0001	< 0.0001
Total	1.6 ± 3.0	8.5 ± 7.4	23.9 ± 8.3	< 0.0001	< 0.0001
Medication use category (0 = none, 1 = peripheral, 2 = central, 3 = opioid)					
	0 ± 0	0.3 ± 0.7	1.1 ± 1.0	< 0.0001	0.03
McGill Pain Questionnaire					
Sensory	0.3 ± 0.8	5.3 ± 4.4	8.4 ± 5.5	< 0.0001	0.008
Affective	0.07 ± 0.3	1.3 ± 1.8	2.1 ± 2.8	< 0.0001	0.13
Total	0.4 ± 1.1	6.6 ± 5.5	10.6 ± 7.1	< 0.0001	0.006
Positive and Negative Affective Scale (PANAS)					
Positive Affect	34.8 ± 8.6	33.4 ± 8.1	28.0 ± 7.7	0.0002	0.002
Negative Affect	12.7 ± 4.6	16.4 ± 7.1	18.1 ± 5.8	< 0.0001	0.25

### Diffusion Tensor Imaging

MRI scanning was performed at multiple sites using different scanner technologies (3T Siemens Trio (NWU and UCLA), 3T Phillips Ingenia (UM), 3T Philips Achieva (UAB), and 3T GE Discovery (SU)). Trans-MAPP neuroimaging data was collected, quality controlled and archived according to multi-site imaging procedures developed collaboratively between the MAPP Research Network, the UCLA PAIN repository and the UCLA Laboratory of Neuroimaging (LONI).

### Data Collection

The data were obtained through the MAPP Network’s central, multicenter collaborative study, the Trans-MAPP Epidemiology/Phenotyping Study (EPS) [1, 16]. Patients and HCs participated in study visits that included extensive phenotyping, collection of biospecimens (blood and urine), and neuroimaging. Extensive phenotype data included information on the duration of symptoms, intensity, and locations of pain (Short Form McGill Pain Questionnaire and body map), medication history, presence of urological symptoms (e.g., urgency, frequency, etc.), and psychological comorbid symptoms (e.g., depression and anxiety, HADS, etc.). Symptom metrics included: symptom duration, American Urological Association (AUA) baseline symptom score index over the past month, baseline Brief Pain Inventory (PAIN) pain severity score and pain interference score, baseline Positive And Negative Affect Schedule (PANAS) positive and negative affect, baseline Genitourinary Pain Index (GUPI) pain score and urinary subscale and QOL impact score and total score, baseline Interstitial Cystitis Symptoms Index (ICSI) and Problem Index (ICPI), SYM-Q Scan: Pain, GUPI ScanL pain subscale and urinary subscale and QOL impact and total score.

MRI scanning was performed at multiple sites using different scanner technology (3T Siemens Trio (NWU and UCLA), 3T Phillips Ingenia (UM), 3T Philips Achieva (UAB), and 3T GE Discovery (SU)). Trans-MAPP neuroimaging data was collected, quality controlled and archived according to multi-site imaging procedures developed collaboratively between the MAPP Research Network, the UCLA PAIN repository and the UCLA Laboratory of Neuroimaging. Detailed procedures and description of the repository are available at PAINrepository.org. All sites were required to complete and pass a site qualification including a set of pilot scans of a human volunteer; the initial scans were reviewed for quality control by the UCLA site, and recommendations and adjustments were made as necessary prior to commencement of study scans. A high resolution structural image was acquired from each subject with a magnetization-prepared rapid gradient-echo (MP-RAGE) sequence, repetition time (TR) = 2200 ms, echo time (TE) = 3.26 ms, slice thickness = 1 mm, 176 slices, 256 x 256 voxel matrices, and 1^3^ mm voxel size. Before entering the scanner, subjects were asked to empty their bladder.

The current study used data exclusively from the NWU and UCLA MAPP Network sites for the following reasons: 1) UM did not acquire DTI data; 2) UAB acquired DTI data with low angular resolution (33 directions); 3 SU only acquired DTI data on female participants. Additionally, UCLA and NWU both used the same scanner model (Siemens Trio 3T) and DTI acquisition parameters were relatively homogeneous. Specifically, the DTI acquisition parameters included: 60–64 directions with *b* = 1000 s/mm^2^ along with 8 *b* = 0 s/mm^2^ images, echo time/repetition time (TE/TR) = 83-88ms/8500-9500ms, a field of view (FOV) of 256mm, a slice thickness of 2mm (no interslice gap), and a matrix of 128x128, resulting in DTI data with 2mm isotropic resolution. [When accounting for site as a covariate in our analyses (see Statistical Parameter Mapping section), it is important to note that few significant site differences were observed between UCLA and NWU within UCPPS vs. HC comparisons (**S2 Fig**).]

### Data Analysis

All DWI scans were processed using eddy-current and motion correction native to the FSL package. FA, MD, and TD images were then calculated using the *MRtrix* package (Brain Research Institute, Melbourne, Australia, http://www.brain.org.au/software/, [27]). GA images were calculated using positive-definite symmetric fourth order tensor matrices calculated via the *fanDtasia* software implemented in MATLAB (http://www.cise.ufl.edu/~abarmpou/lab/fanDTasia/), based on previous publications involving calculations of higher-order tensors [28–30], and implemented in a previous study of high-order DTI in glioblastoma [31]. TD images were calculated using the following steps for each subject: 1) a brain mask was created using the *b* = 0 s/mm^2^ image; 2) a tensor fit was applied to the DTI data, and FA and MD images were created; 3) the FA image was used to create a high FA white matter mask by thresholding the FA image at 0.7 and eroding the resulting mask with three passes to obtain a response function for single-fiber-orientation voxels; 4) calculation of the spherical harmonic response function using constrained spherical deconvolution (CSD) [32] of the DWI data and a maximum harmonic order (*l*_*max*_) of 8, the maximum attainable with the number of parameters required (45); 5) visual inspection was performed for each white matter mask and response function; 6) application of whole brain probabilistic tractography [33] using the 2^nd^ order integration over fiber orientation distributions (iFOD2) algorithm [34] by seeding 1 million voxels randomly throughout the brain with track stopping criteria of an FA of 0.1, minimum curvature radius of 1mm, and minimum track length of 10mm; and 7) calculating TD images at 0.5mm isotropic resolution by counting the number of fiber tracts passing through each image voxel after probabilistic tractography.

### Statistical Parameter Mapping

FA images for each subject were registered to the Johns Hopkins University DTI atlas (ICBM-DTI-81 1mm FA atlas) using a 12-degree of freedom linear affine transformation using FSL. After linear registration, elastic (nonlinear) registration was performed between individual FA maps and the ICBM-DTI-81 FA atlas using the FNIRT command in FSL. The transformation matrices (linear, then nonlinear) were then used to align the other scalar metrics (MD, eigenvalues, and TDIs) to the same atlas space.

Voxelwise comparisons were performed in a white matter mask (FA > 0.3) that also included deep gray matter structures such as the basal ganglia and the thalamus, as outlined previously [10]. Statistical parametric maps (SPM) were created using a voxel-wise *t*-test with covariates that included age, site, gender, body mass index, and phenotype data (i.e. symptom duration, intensity, locations, medical history, presence of urological symptsoms, and psychological comorbid symptoms. (Note that when testing for voxelwise differences between genders for each group, gender was excluded from the covariate file). The SPMs were created using AFNI’s *3dttest++* function, thresholded at a level of significance, *P* < 0.05. For each test, a cluster threshold was applied from permutation testing as follows: 1) the groups were split and randomized 1000 times, keeping a list of the sizes of the significantly different clusters; then 2) the 95^th^ percentile of the list of cluster sizes above 20 μL was used as the cluster threshold for each particular test. The resultant cluster sizes were around 100 μL, which is less than (but still comparable to) a more conservative estimate of a 250 μL minimum cluster size as used in a previous study [10] based on permutation calculations outlined by Bullmore *et al* [35]. T-tests and clustering were performed between HC and UCPPS, IBS and UCPPS, and between males and females for HC, IBS, and UCPPS.

### Correlations of DTI and TDI Measurements with Symptom Scores

In order to explore potential relationships between imaging parameters and symptoms in patients with UCPPS we examined regions that were significantly different between UCPPS subjects and HC, extracted the mean value (for each metric), and then calculated the correlation coefficient between these imaging metrics and a variety of symptom variables. Potential trends in the data were visualized using a correlation matrix or “heat-map” containing the Pearson correlation coefficients for each image contrast and symptom score. Since this was a hypothesis generating exploratory analysis only, no corrections for multiple comparisons were performed. Rows in the correlation matrix were sorted by calculation of a pairwise distance function for symptom score correlations between the different ROIs, and then performing agglomerative hierarchical clustering. Dendrograms were generated based on the resulting clustering and used for interpretation of possible associations with clinical information.

## Results

### Clinical Parameters

No difference in age or BMI was observed between HCs, IBS, and UCPPS subjects (**Table 1** *; P = 0*.*31 and P = 0*.*42*, respectively); however, differences in both anxiety and depression symptom scores were observed between patients groups (*P<0*.*0001*), with UCPPS showing significantly higher scores compared with IBS or HCs (*UCPPS vs*. *IBS*, P = 0.03 for anxiety scale and P = 0.003 for depression). As expected, the genitourinary pain index (GUPI) was significantly different between groups (*P<0*.*0001*) with UCPPS showing significantly worse pain compared with the other groups (*P<0*.*0001*). Group differences were also noted for the McGill Pain Questionnaire (*P<0*.*0001*) and Positive (*P = 0*.*002*) and Negative Affective Scale (PANAS) (*P<0*.*0001*), with UCPPS patients exhibiting significantly higher levels of sensory pain on the McGill Pain Questionnaire (*P = 0*.*008*), total McGill Pain score (*P = 0*.*006*), and significantly lower levels of positive affect on the PANAS scale (*P = 0*.*002*).

### Anatomical Comparisons Between UCPPS Patients and HCs

**Fig 1:**
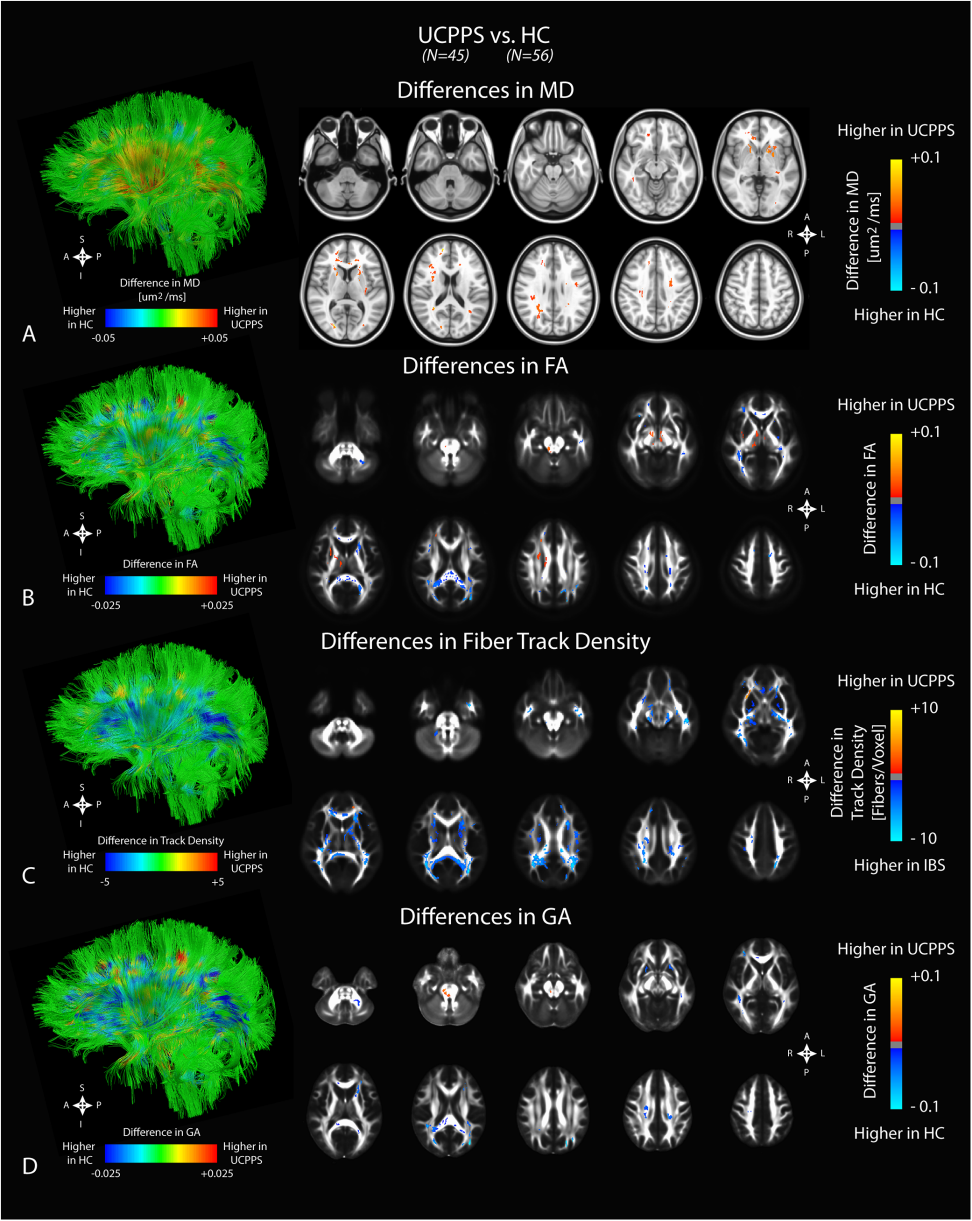
Anatomical localization of significant differences in DTI and TDI measurements between UCPPS patients (*N = 45*) and HCs (*N = 56*). A) Observed differences in mean diffusivity (MD). B) Observed differences in fractional anisotropy (FA). C) Observed differences in fiber track density. D) Observed differences in generalized anisotropy (GA). Significant clusters were determined by thresholding based on level of statistical significance (*P < 0*.*05*) and cluster-based corrections using random permutation analysis. Left column illustrates differences projected onto representative white matter fiber tracts.

In order to determine whether widespread microstructural reorganization is present in the brain of UCPPS subjects we first compared regional differences in DTI and TDI metrics between UCPPS patients and HCs. After correction for multiple covariates including age, site, body mass index, and both clinical and symptom scores, DTI and TDI revealed distinct anatomical regions that differed significantly between UCPPS patients and HCs. Generally, MD measurements tended to be higher in UCPPS patients compared with HCs (**Table 2**). In particular, we observed a higher MD in the basal ganglia, areas of crossing white matter fibers in the right frontal lobe, association fibers and right superior and posterior corona radiata fibers, corona radiata fibers on the left hemisphere, and areas within the genu of the corpus callosum (**Fig 1A**). When examining FA, most regions were significantly *lower* in UCPPS compared to HCs (**Table 3**). Specifically, the genu and splenium of the corpus callosum exhibited large areas of lower FA in UCPPS compared with HC. Interestingly, regions in and adjacent to the thalamus and basal ganglia showed an *increase* in FA in UCPPS compared with HC, as did a segment in the left somatosensory cortex consistent with regions associated with pelvic somatosensory integration (**Fig 1B**). Results from TDI comparisons suggest fiber track density was significantly lower in UCPPS patients compared with HCs in a large area of the brain, traversing numerous anatomical locations thought to be involved in chronic pain perception (**Table 4**). These areas included regions of thick white matter in both the genu and splenium of the corpus callosum, areas adjacent to deep gray matter structures, and bilateral fibers connecting parietal regions (**Fig 1C**). Similarly to FA, GA maps demonstrated large areas of decreased anisotropy, with the corpus callosum and some of the corona radiata being notable examples (**Table 5**). However, in the GA results we did not see the increased anisotropy in the deep gray matter or somatosensory cortex that was seen in the FA analysis; however, there was increased GA in brain stem regions of UCPPS subjects, which is also reflected in the FA results (**Fig 1D**). Together, these results support the hypothesis that UCPPS compared to HCs is associated with relatively widespread microstructural reorganization within the brain.

**Table 2:** Anatomical regions and corresponding cluster volumes showing significant differences in MD between UCPPS patients and HCs (HCs). Minimum cluster size = 171 uL from permutation analysis.

ROI	Anatomic Regions	Cluster Volume [uL]	UCPPS vs. HC
1	Lt. Basal Ganglia, Putamen	2394	Higher MD in UCPPS
2	Rt. Posterior Coronal Radiata	1334	Higher MD in UCPPS
3	Rt. Sensorimotor Integration Fibers	795	Higher MD in UCPPS
4	Lt. Sensorimotor Integration Fibers	713	Higher MD in UCPPS
5	Rt. Anterior Cingulate	700	Higher MD in UCPPS
6	Rt. Forceps Minor, Genu Corpus Callosum	673	Higher MD in UCPPS
7	Rt. External Capsule	632	Higher MD in UCPPS
8	Lt. Posterior Sup. Long. Fasciculus	455	Higher MD in UCPPS
9	Rt. Inferior Longitudinal Fasciculus	418	Higher MD in UCPPS
10	Rt. Internal Capsule	403	Higher MD in UCPPS
11	Lt. Precentral Gyrus Association Fibers	336	Higher MD in UCPPS
12	Lt. Inferior Longitudinal Fasciculus	315	Higher MD in UCPPS
13	Rt. Basal Ganglia, Putamen	288	Higher MD in UCPPS
14	Lt. Basal Ganglua, Globus Palladis	266	Higher MD in UCPPS
15	Rt. Anterior Cingulate	259	Higher MD in UCPPS
16	Rt. Prefrontal White Matter Projections	253	Higher MD in UCPPS
17	Lt. Forceps Minor, Genu Corpus Callosum	243	Higher MD in UCPPS
18	Lt. Inferior Longitudinal Fasciculus	241	Higher MD in UCPPS
19	Rt. Inferior Longitudinal Fasciculus	215	Higher MD in UCPPS
20	Lt. Posterior Sup. Long. Fasciculus	207	Higher MD in UCPPS
21	Genu of Corpus Callosum	206	Higher MD in UCPPS
22	Splenium of Corpus Callosum	176	Higher MD in UCPPS

**Table 3:** Anatomical regions and corresponding cluster volumes showing significant differences in FA between UCPPS patients and HCs (HCs). Minimum cluster size = 111 uL from permutation analysis.

ROI	Anatomic Regions	Cluster Volume [uL]	UCPPS vs. HC
1	Splenium of Corpus Callosum	1373	Higher FA in HC
2	Splenium of Corpus Callosum	458	Higher FA in HC
3	Lt. Sensorimotor Integration Fibers	412	Higher FA in UCPPS
4	Rt. Inf. Longitudinal Fasciculus	390	Higher FA in HC
5	Rt. Forceps Major, Splenium Corpus Callosum	352	Higher FA in HC
6	Lt. Internal Capsule	338	Higher FA in UCPPS
7	Splenium of Corpus Callosum	331	Higher FA in HC
8	Lt. Forceps Major, Splenium Corpus Callosum	271	Higher FA in HC
9	Inf. Genu Corpus Callosum	263	Higher FA in HC
10	Lt. Cerebellar Peduncle	258	Higher FA in HC
11	Genu Corpus Callosum	258	Higher FA in HC
12	Splenium Corpus Callosum	252	Higher FA in HC
13	Lt. Temporoparietal Fibers	248	Higher FA in HC
14	Rt. Thalamus, Prefrontal Projections	243	Higher FA in UCPPS
15	Rt. Prefrontal Projections	237	Higher FA in HC
16	Rt. Inf. Longitudinal Fasciculus	205	Higher FA in HC
17	Rt. Midbrain, Reticular Formation	181	Higher FA in UCPPS
18	Lt..Uncinate Fasciculus	170	Higher FA in HC
19	Rt. Midbrain, Medial Lemniscus	169	Higher FA in UCPPS
20	Lt. Temporoparietal Fibers	149	Higher FA in HC
21	Lt. Substantia Nigra	146	Higher FA in UCPPS
22	Rt. Sup. Longitudinal Fasciculus	138	Higher FA in HC
23	Lt. Inferior Longitudinal Fasciculus	133	Higher FA in HC
24	Rt. External Capsule	120	Higher FA in UCPPS
25	Lt. Posterior Coronal Radiata	119	Higher FA in HC
26	Rt. Basal Ganglia, Putamen	117	Higher FA in UCPPS
27	Lt. M1 Association Fibers	117	Higher FA in HC
28	Rt. Basal Ganglia, Striatum	116	Higher FA in UCPPS
29	Lt. Midbrain, Medial Lemniscus	114	Higher FA in UCPPS
30	Lt. Posterior Cingulate Bundle	111	Higher FA in HC

**Table 4:** Anatomical regions and corresponding cluster volumes showing significant differences in fiber track density on TDI between UCPPS patients and HCs (HCs). Minimum cluster size = 111 uL from permutation analysis.

ROI	Anatomic Regions	Cluster Volume [uL]	UCPPS vs. HC
1	Genu and Splenium of Corpus Callosum,	31815	Higher Track Density in HC
	Bilateral Coronal Radiata, Bilateral Cingulate,		
	Bilateral Sup. Long. Fasciculus, Bilateral Inf. Long. Fasciculus		
2	Lt. Superior Longitudinal Fasciculus	894	Higher Track Density in HC
3	Lt. Basal Ganglia, Striatum, Putamen	768	Higher Track Density in HC
4	Lt. Inf. Longitudinal Fasciculus	503	Higher Track Density in HC
5	Lt. Forceps Minor, Genu Corpus Callosum	406	Higher Track Density in HC
6	Rt. Sensorimotor Integration Fibers	379	Higher Track Density in HC
7	Rt. Posterior Inf. Longitudinal Fasciculus	326	Higher Track Density in HC
8	Lt. Posterior Inf. Longitudinal Fasciculus	302	Higher Track Density in HC
9	Lt. Superior Longitidnal Fasciculus	289	Higher Track Density in HC
10	Lt. Anterior Inf. Longitudinal Fasciculus	284	Higher Track Density in HC
11	Rt. Basal Ganglia, Globus Pallidus	264	Higher Track Density in HC
12	Rt. Prefrontal Projections	246	Higher Track Density in HC
13	Rt. Midbrain, Corticospinal Tracts	234	Higher Track Density in HC
14	Rt. Sup. Longitidual Fasciculus	206	Higher Track Density in HC
15	Rt. Anterior Inf. Longitudinal Fasciculus	205	Higher Track Density in HC
16	Lt. Cerebral Peduncle	187	Higher Track Density in HC
17	Rt. Lateral Forceps Minor, Genu Corpus Callosum	171	Higher Track Density in HC
18	Lt. Posterior Coronal Radiata, Corticospinal Tracts	164	Higher Track Density in HC
19	Rt. Posterior Inf. Longitudinal Fasciculus	159	Higher Track Density in HC
20	Rt. Thalamus, Posterior Parietal Projections	158	Higher Track Density in HC
21	Rt. Exterman Capsule	155	Higher Track Density in HC
22	Rt. Inf. Longitudinal Fasciculus	131	Higher Track Density in HC
23	Rt. Anterior Cingulate	130	Higher Track Density in HC
24	Rt. Prefrontal Projections	117	Higher Track Density in HC

**Table 5:** Anatomical regions and corresponding cluster volumes showing significant differences in GA between UCPPS patients and HCs (HCs). Minimum cluster size = 117 uL from permutation analysis.

ROI	Anatomic Regions	Cluster Volume [uL]	UCPPS vs. HC
1	Rt. Forceps Major, Splenium of Corpus Callosum	1165	Higher GA in HC
2	Lt. Ant. Corona Radiata	503	Higher GA in HC
3	Genu of Corpus Callosum	451	Higher GA in HC
4	Lt. Post. and Sup. Corona Radiata	449	Higher GA in HC
5	Splenium Corpus Callosum	420	Higher GA in HC
6	Rt. Posterior Thalamic Radiation	356	Higher GA in HC
7	Rt. Sup. Corona Radiata	352	Higher GA in HC
8	Lt. Forceps Major	339	Higher GA in HC
9	Rt. Corticospinal Tracts Brain Stem	334	Higher GA in UCPPS
10	Lt. Sup. Longitudinal Fasciculus	265	Higher GA in HC
11	Rt. Inf. Fronto-Occipital Fasciculus	259	Higher GA in HC
12	Rt. Sup. Corona Radiata	242	Higher GA in HC
13	Lt. Sup. Longitudinal Fasciculus	212	Higher GA in HC
14	Lt. Forceps Major, Splenium of Corpus Callosum	196	Higher GA in HC
15	Lt. Inf. Fronto-Occipital Fasciculus	193	Higher GA in HC
16	Lt. Ant. Thalamic Radiation	191	Higher GA in HC
17	Rt. Sup. Longitudinal Fasciculus	185	Higher GA in HC
18	Splenium of Corpus Callosum	168	Higher GA in HC
19	Lt. Putamen	166	Higher GA in HC
20	Lt. Temporoparietal Fibers	151	Higher GA in HC
21	Rt. Putamen	146	Higher GA in HC
22	Rt. Post. Thalamic Radiation	144	Higher GA in HC
23	Lt. Mid. Cerebellar Peduncle	122	Higher GA in HC
24	Lt. Temporoparietal Fibers	120	Higher GA in HC
25	Lt. Inf. Fronto-Occipital Fasciculus	118	Higher GA in HC

### Anatomical Comparisons Between UCPPS Patients and IBS Patients

**Fig 2:**
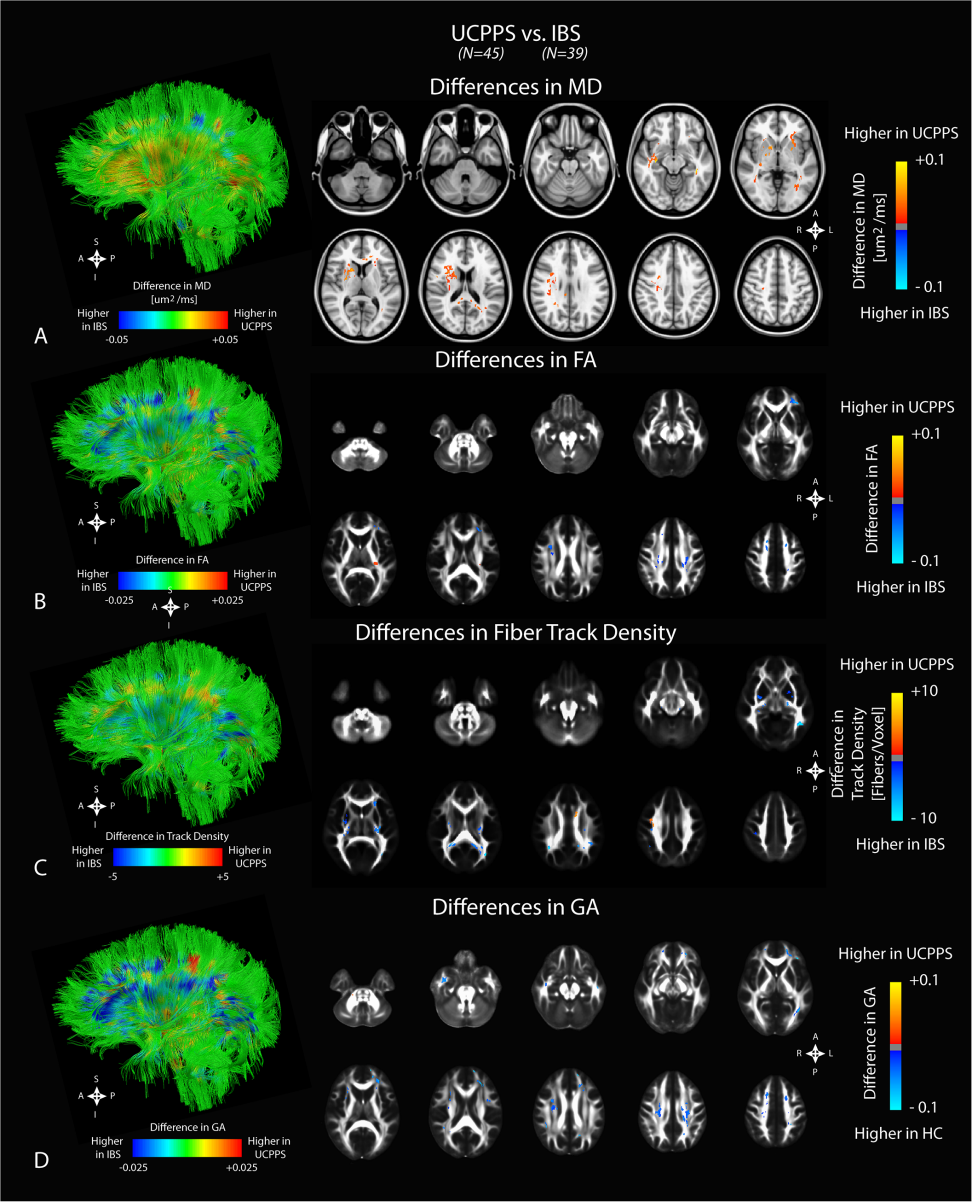
Anatomical localization of significant differences in DTI and TDI measurements between UCPPS patients (*N = 45*) and positive control patients with IBS (*N = 39*). A) Observed differences in mean diffusivity (MD). B) Observed differences in fractional anisotropy (FA). C) Observed differences in fiber track density. D) Observed differences in generalized anisotropy (GA). Significant clusters were determined by thresholding based on level of statistical significance (*P < 0*.*05*) and cluster-based corrections using random permutation analysis. Left column illustrates differences projected onto representative white matter fiber tracts.

To identify changes specific to UCPPS that are not generally associated with other chronic visceral pain syndromes, DTI and TDI measurements were compared between patients with UCPPS and IBS. Examination of MD suggested patients with UCPPS had significantly higher MD compared with IBS patients, particularly in areas of the basal ganglia, right periventricular white matter, temporal lobe projections, and fibers projecting to the right primary motor cortex along with association fibers in the frontal regions of the right hemisphere (**Fig 2A**). Analysis of regional FA differences between UCPPS and IBS conditions indicated that UCPPS had lower FA in a region within the splenium of the corpus callosum, within the superior portion of the right corona radiata, as well as a small region near the insula (**Fig 2B**). Interestingly, there were a few regions with *increased* FA in association fibers within the frontal lobe. Track density measurements were compared between UCPPS and IBS patients. As expected, results suggest the differences in track density between these chronic pain conditions were not as pronounced as those observed between UCPPS and HCs. A region in the genu of the corpus callosum appeared to have a higher track density in UCPPS patients, suggesting a higher degree of connectivity within the frontal lobe in patients with UCPPS compared with patients exhibiting symptoms of IBS, whereas a region in the splenium of the corpus callosum demonstrated a higher track density in patients with IBS compared with UCPPS. Additionally, we observed regions of lower track density in the basal ganglia, adjacent white matter regions, and bilateral parietal regions in patients with UCPPS compared with IBS, consistent with but smaller in volume than comparisons with HCs. Also, a region in the left frontal lobe near the crossing of the cingulum and corpus callosum appeared to have a higher track density in IBS, as did regions in the right primary motor cortex and right premotor cortex (**Fig 2C**). The regional GA differences between UCPPS and IBS yielded similar patterns to those seen in FA, with distributed decreased GA in white matter regions; however, regions of increased GA in UCPPS subjects were lacking, similar to results shown between UCPPS and HCs (**Fig 2D**).

### Diffusion Signatures for UCPPS Patients

**Fig 3:**
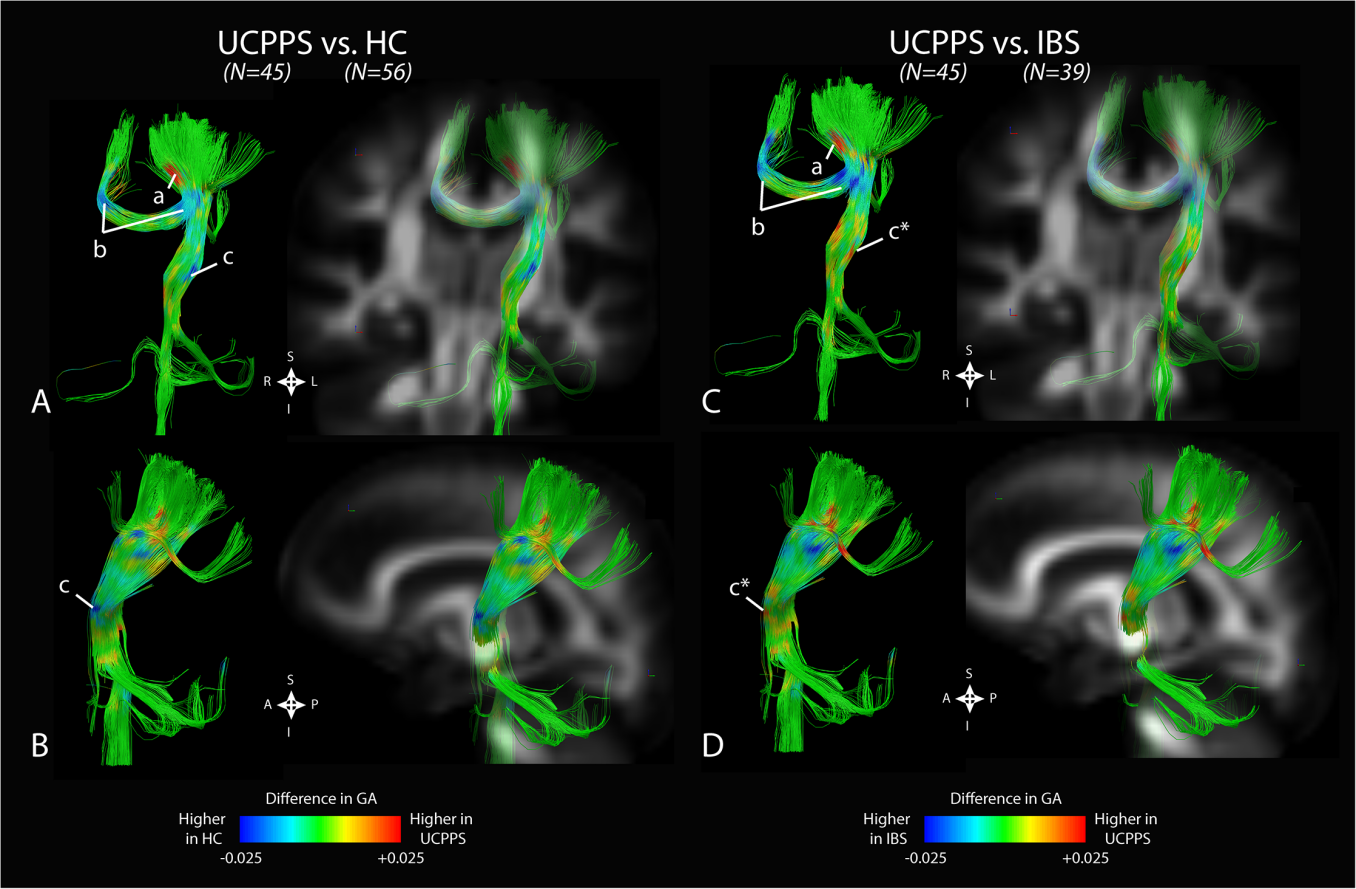
Combined anatomical localization of significant differences in generalized anisotropy (GA) between UCPPS patients and both HCs and IBS patients in primary somatosensory tracts. A) Coronal and B) sagittal views of fiber tracts showing increased GA in regions adjacent to pelvic representation (“*a*”), decreased GA in the splenium of the corpus callosum (“*b*”), and decreased GA near the ventral nuclei of the thalamus (“*c*”) in UCPPS patients compared to healthy controls (HCs). C) Coronal and D) sagittal views of fiber tracts showing increased GA in regions adjacent to pelvic representation (“*a*”), decreased GA in the splenium of the corpus callosum (“*b*”), and *inreased* GA near the ventral nuclei of the thalamus (“c*”) in UCPPS patients compared to IBS patients.

Taken together, results from UCPPS comparisons to both HCs and IBS patients illustrated focal and distinct changes in diffusion anisotropy, localized to the somatosensory pathway (**Fig 3**). Specifically, we observed a region of white matter adjacent to the pelvic homunculus with significantly higher GA (and FA) in UCPPS compared with both HCs and IBS patients (“*a*” in **Fig 3A and 3C**), whereas splenium regions of the corpus callosum demonstrated lower GA (and FA) in UCPPS compared with both groups (“*b*” in **Fig 3A and 3C**). Interestingly, we also noted differences in diffusion anisotropy near the ventral nuclei of the thalamus (“*c*” and “c*” in **Fig 3A–3D**), with UCPPS showing lower GA (and FA) compared with HCs and higher GA (and FA) when compared with IBS patients.

### Sex Differences

**Fig 4:**
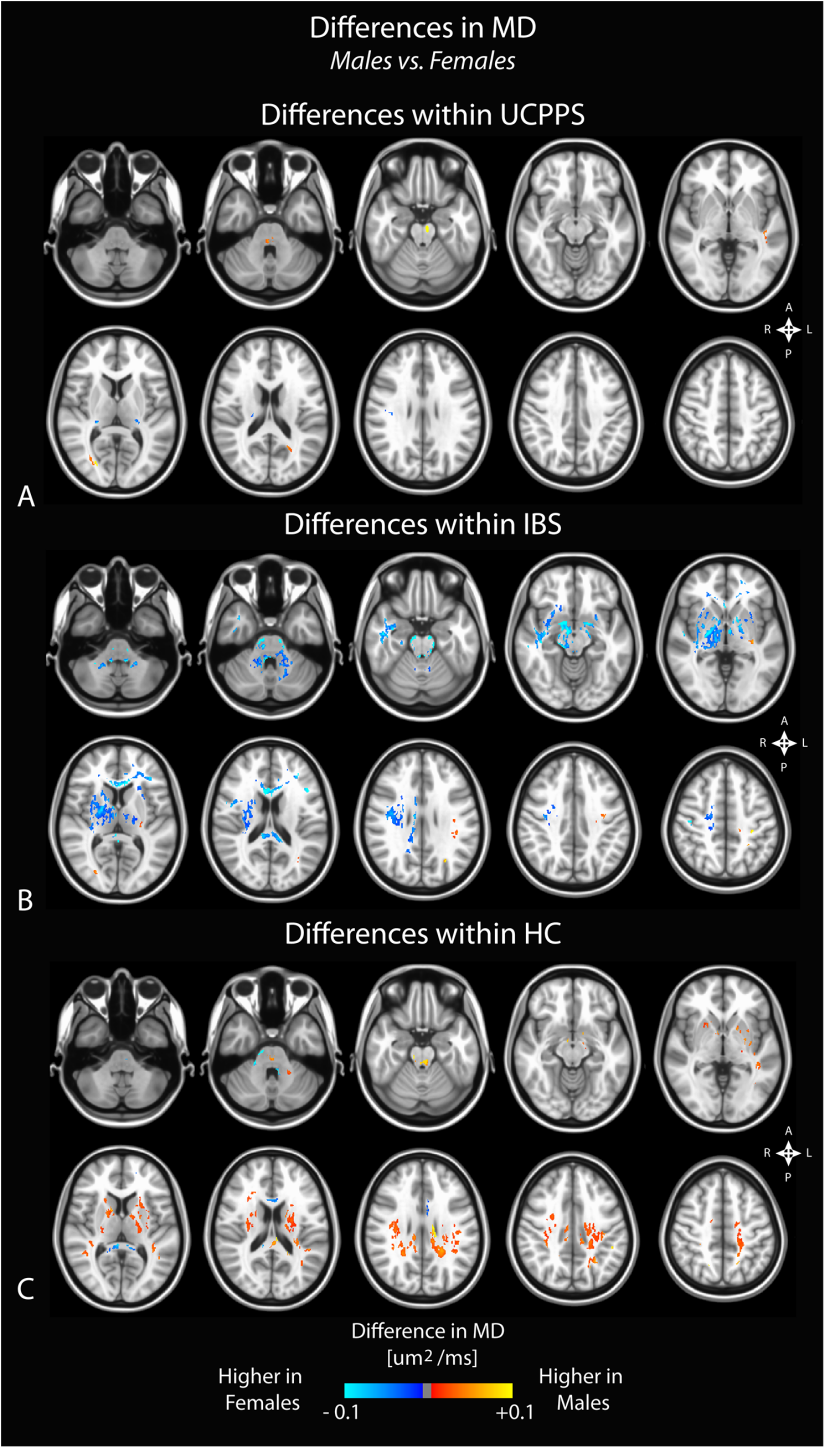
Sex differences in mean diffusivity (MD) within A) UCPPS patients, B) positive control patients with IBS, and C) healthy control (HC) participants. Significant clusters were determined by thresholding based on level of statistical significance (*P < 0*.*05*) and cluster-based corrections using random permutation analysis.

Since many pain conditions have been shown to result in sex-related reorganization within the brain, we compared DTI and TDI metrics between men and women within each of the cohorts (UCPPS, IBS, and HC). Results demonstrated that MD was generally higher in males compared with females within HCs, though a portion of the genu of the corpus callosum appeared to have higher MD in women (**Fig 4A**). Regions within the basal ganglia and surrounding white matter, along with regions in the cingulum, corona radiata, and short association fibers also exhibited a significantly higher MD in males compared with females. Within UCPPS patients, few regions with differences in MD were observed between males and females (**Fig 4B**). Consistent with previous reports [10], male IBS patients appeared to have lower MD compared with females, particularly in the basal ganglia, thalamus, internal capsule, brainstem, corpus callosum and corona radiata. Additionally, a few regions with increased MD within male IBS patients were observed, specifically areas involving the primary motor and sensory cortices (**Fig 4C**).

**Fig 5:**
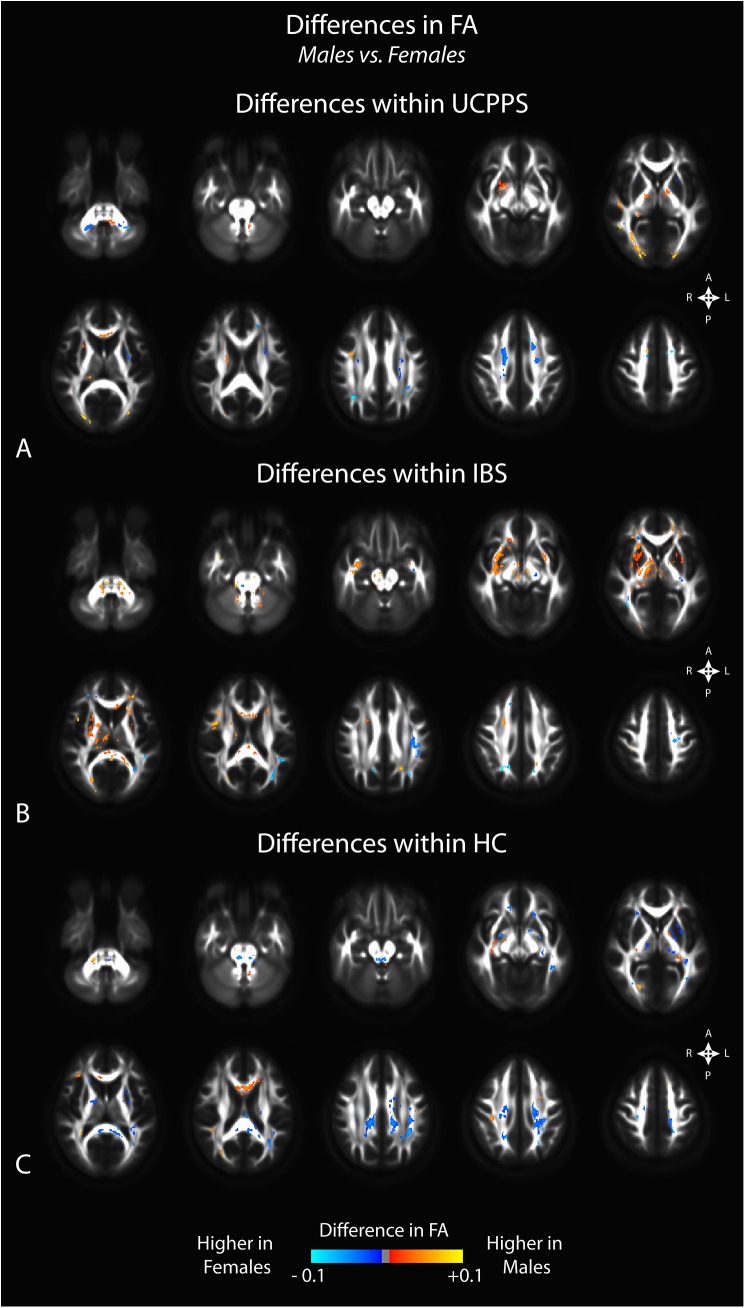
Sex differences in fractional anisotropy (FA) within A) UCPPS patients, B) positive control patients with IBS, and C) healthy control (HC) participants. Significant clusters were determined by thresholding based on level of statistical significance (*P < 0*.*05*) and cluster-based corrections using random permutation analysis.

**Fig 6:**
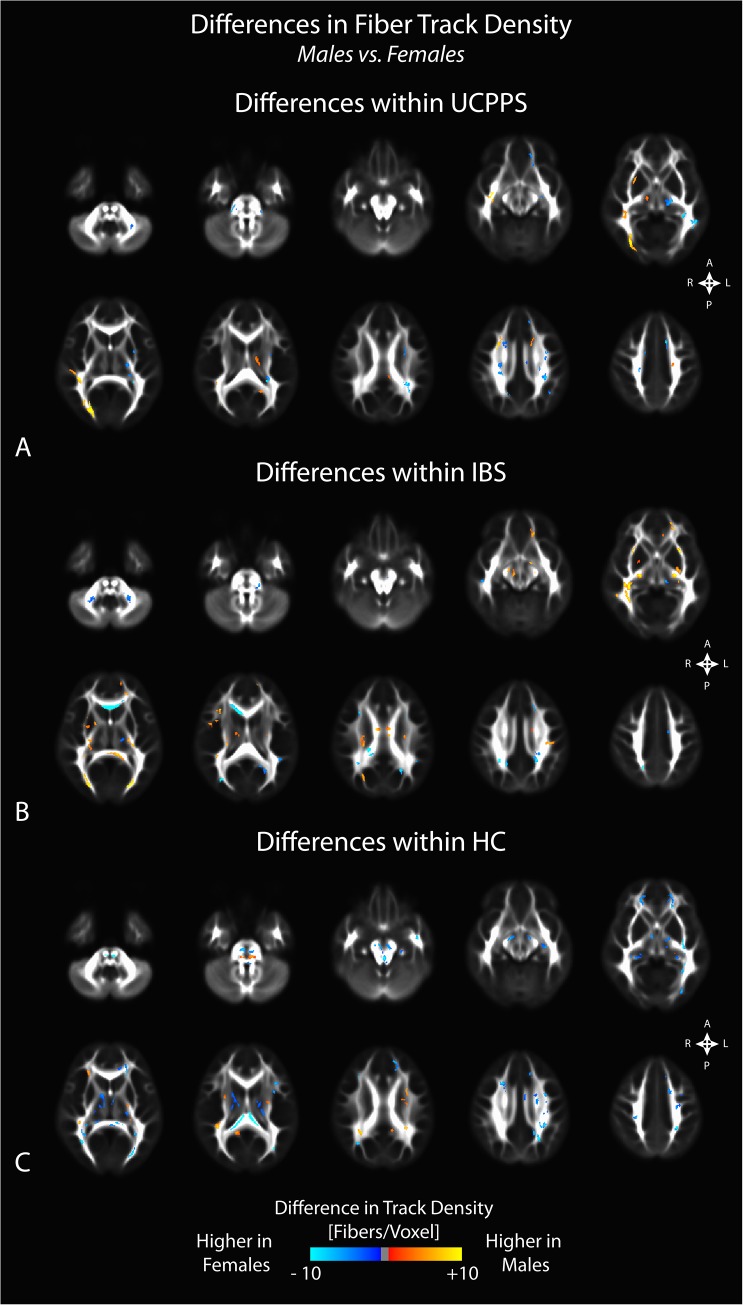
Sex differences in fiber track density within A) UCPPS patients, B) positive control patients with IBS, and C) healthy control (HC) participants. Significant clusters were determined by thresholding based on level of statistical significance (*P < 0*.*05*) and cluster-based corrections using random permutation analysis.

Comparison of regional FA differences between males and females for each patient group indicated similar differences. In particular, female HC subjects had higher FA in the genu of the corpus callosum compared with male HCs, whereas male HCs had higher FA in the splenium and white matter regions generally localized within the left hemisphere (**Fig 5A**). Like changes in MD, we did not observe substantial changes in FA between male and female patients with UCPPS, though we did observe a few regions in the superior regions of the left hemisphere that appeared consistent with, but smaller than, regions observed when comparing male and female HC subjects (**Fig 5B**). Additionally, we observed higher FA in male IBS patients in most regions, especially in thalamic and basal ganglia regions, along with the genu of the corpus callosum (**Fig 5C**). Examination of regional track density measurements between males and females for each patient group suggested a generally higher track density in males compared with females for all groups (**Fig 6**). The specific anatomical regions appeared similar between all three patient groups (UCPPS, IBS, and HC).

**Fig 7:**
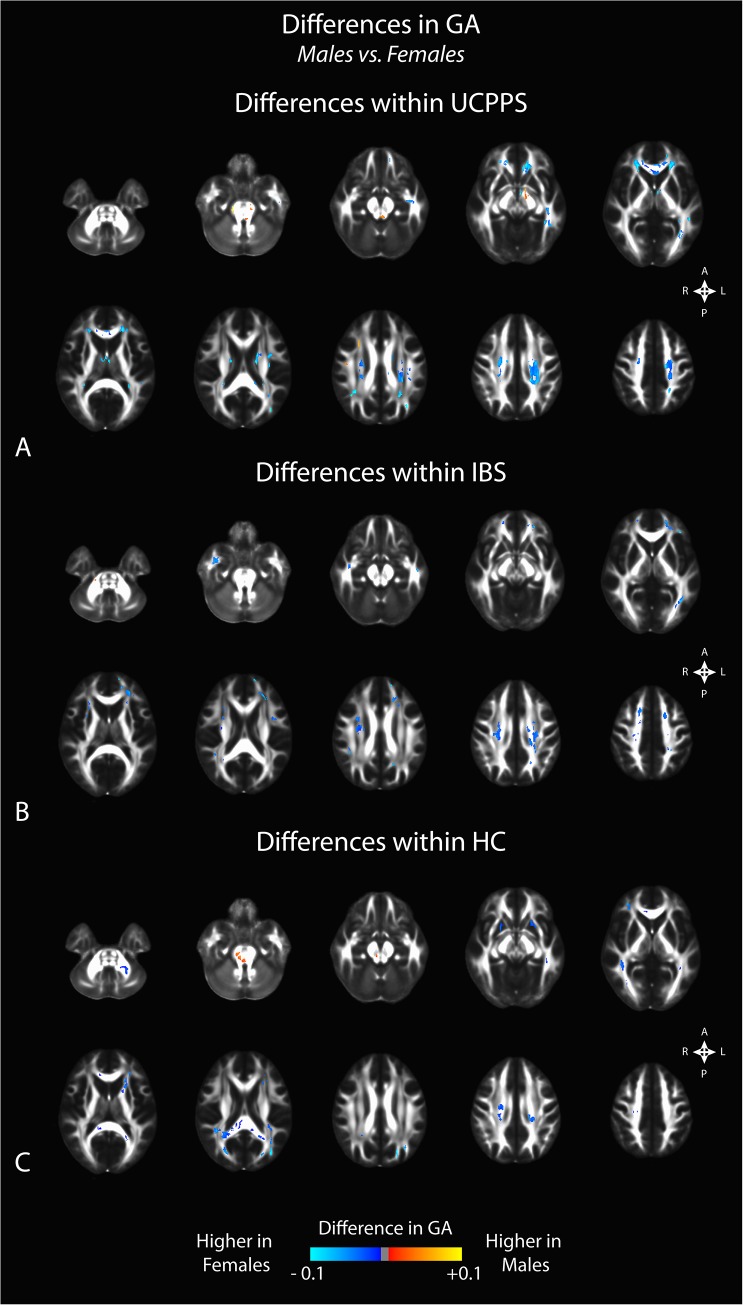
Sex differences in generalized anisotropy (GA) within A) UCPPS patients, B) positive control patients with IBS, and C) healthy control (HC) participants. Significant clusters were determined by thresholding based on level of statistical significance (*P < 0*.*05*) and cluster-based corrections using random permutation analysis.

Results from the GA comparisons demonstrated similar trends to the results demonstrated by FA, but in many cases appeared more drastic (**Fig 7**). Overall area of significantly different voxels increased in the GA analysis, with UCPPS males showing lower GA, IBS males showing higher GA than females, and HC males showing a mix of regions of higher GA and regions of lower GA than females. Again, UCPPS subjects represented the least widespread changes between males and females.

Together, these results suggest sex-differences in microstructural organization within the brain exist and that the specific differences are unique within the various chronic pain syndromes and when compared with HCs.

### Correlations with Symptom Scores

**Fig 8:**
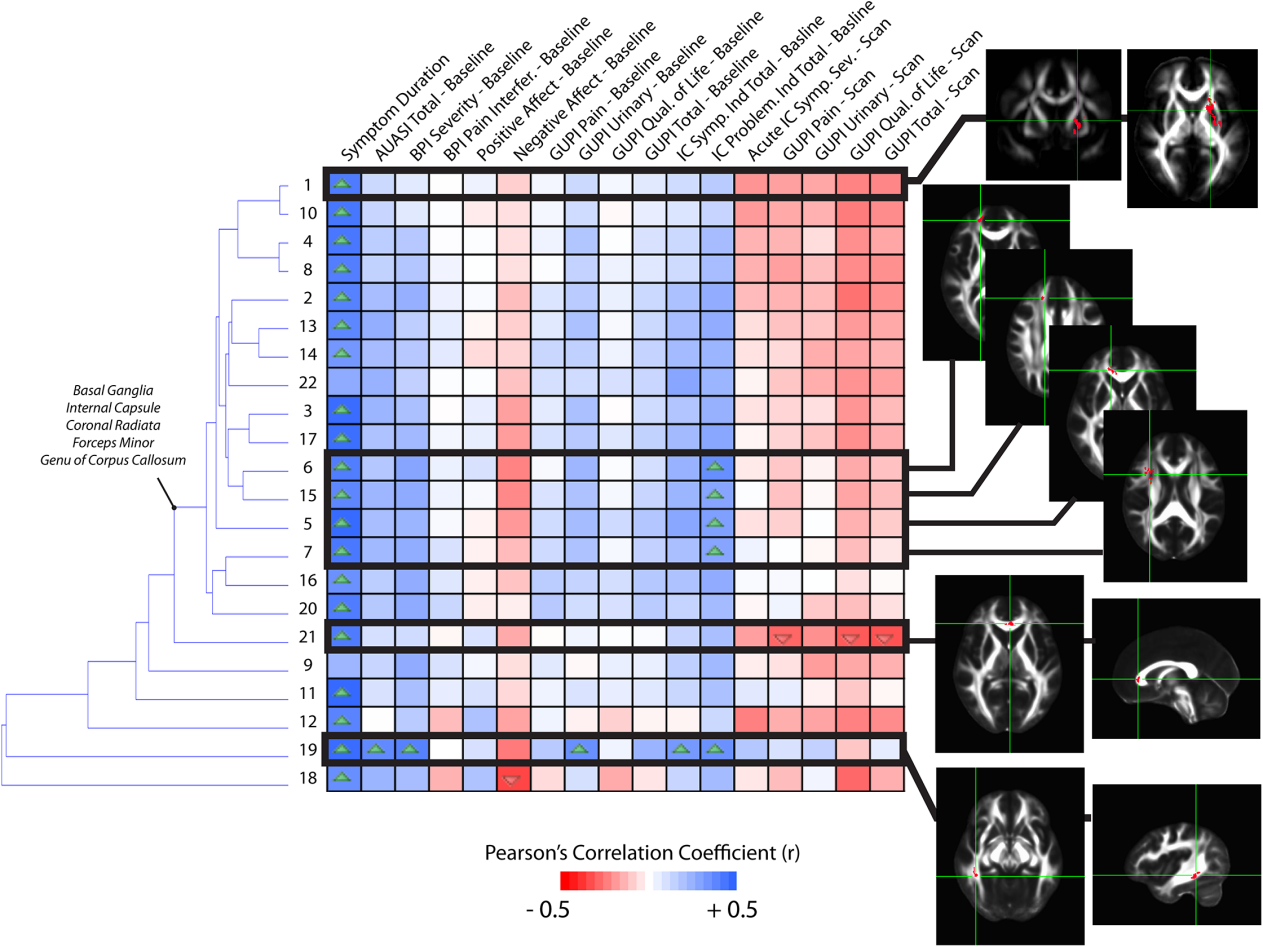
Correlation matrix between mean MD measurements and MAPP symptom scores in UCPPS patients, localized to ROIs identified as different between UCPPS and HCs on statistical parameter maps. Dendrograms on the left side of the correlation matrix show hierarchical clustering of ROIs based on the association between MD measurements and symptom scores. Images showing ROI localization are chosen for select associations. Up arrows within cells denote significantly positive correlations (*P<0*.*05*) and down arrows within cells denote significantly negative correlations (*P<0*.*05*). (Note the level of significance was not corrected for multiple comparisons in this exploratory analysis).

**Fig 9:**
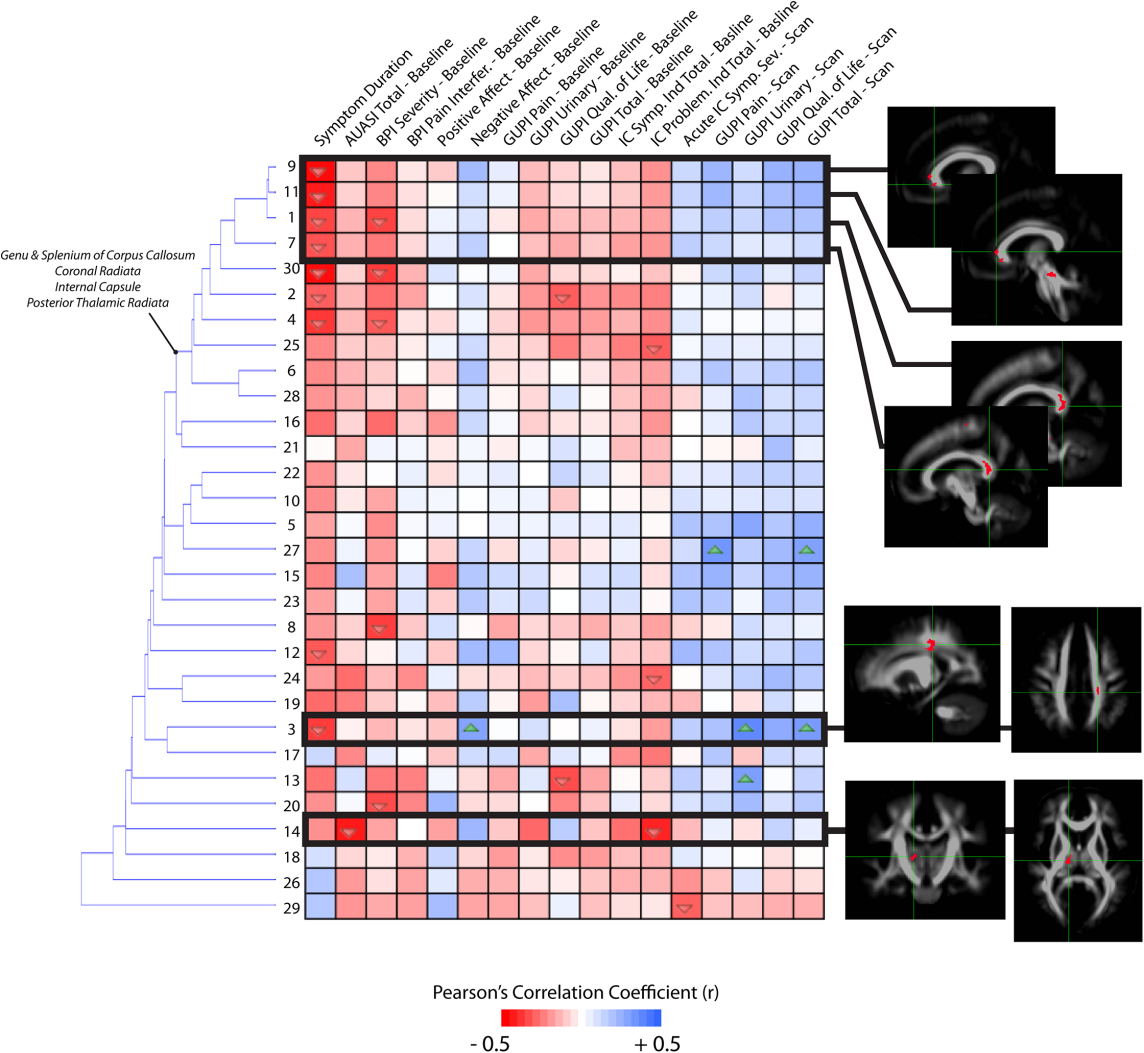
Correlation matrix between mean FA measurements and MAPP symptom scores in UCPPS patients, localized to ROIs identified as different between UCPPS and HCs on statistical parameter maps. Dendrograms on the left side of the correlation matrix show hierarchical clustering of ROIs based on the association between FA measurements and symptom scores. Images showing ROI localization are chosen for select associations. Up arrows within cells denote significantly positive correlations (*P<0*.*05*) and down arrows within cells denote significantly negative correlations (*P<0*.*05*). (Note the level of significance was not corrected for multiple comparisons in this exploratory analysis).

**Fig 10:**
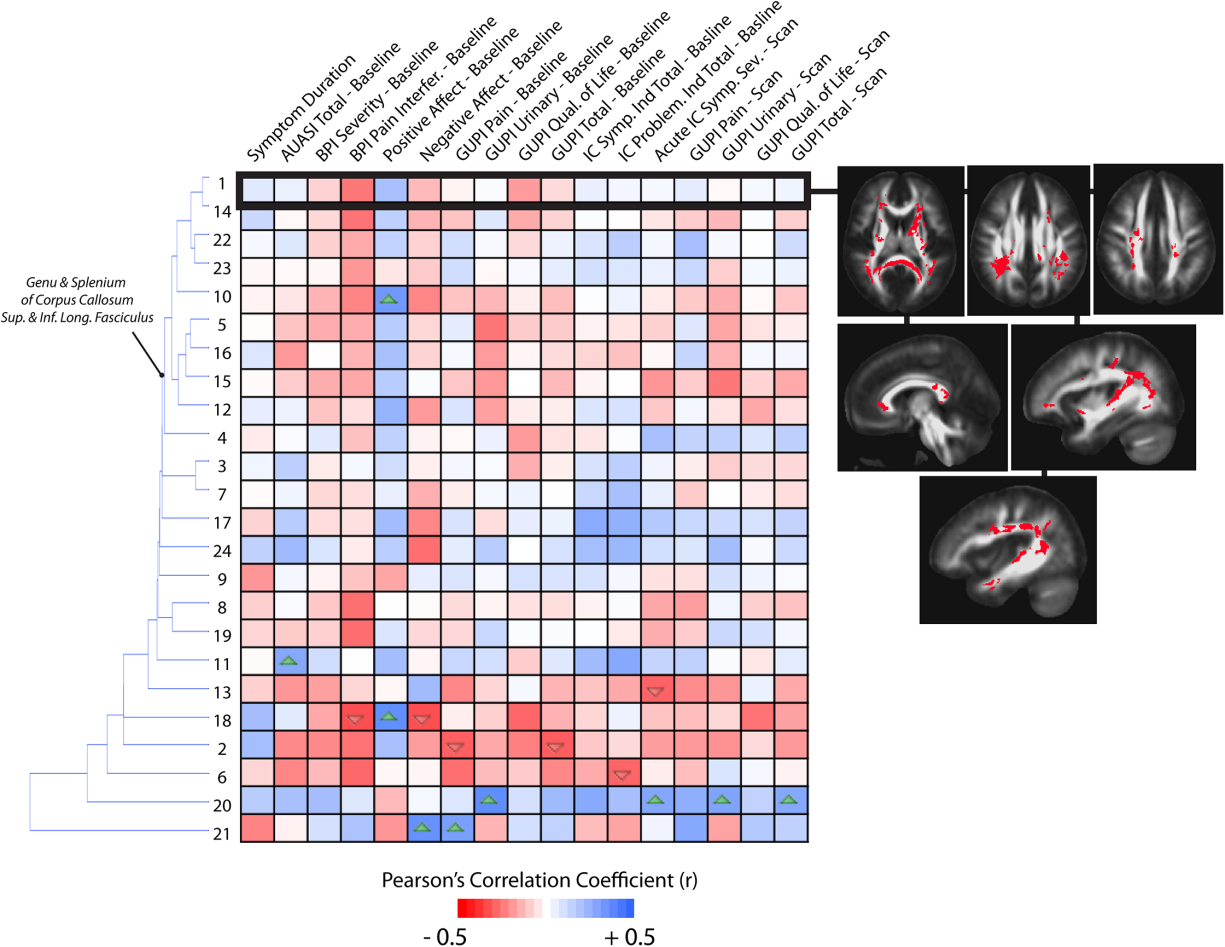
Correlation matrix between mean track density measurements and MAPP symptom scores in UCPPS patients, localized to ROIs identified as different between UCPPS and HCs on statistical parameter maps. Dendrograms on the left side of the correlation matrix show hierarchical clustering of ROIs based on the association between track density measurements and symptom scores. Images showing ROI localization are chosen for select associations. Up arrows within cells denote significantly positive correlations (*P<0*.*05*) and down arrows within cells denote significantly negative correlations (*P<0*.*05*). (Note the level of significance was not corrected for multiple comparisons in this exploratory analysis).

**Fig 11:**
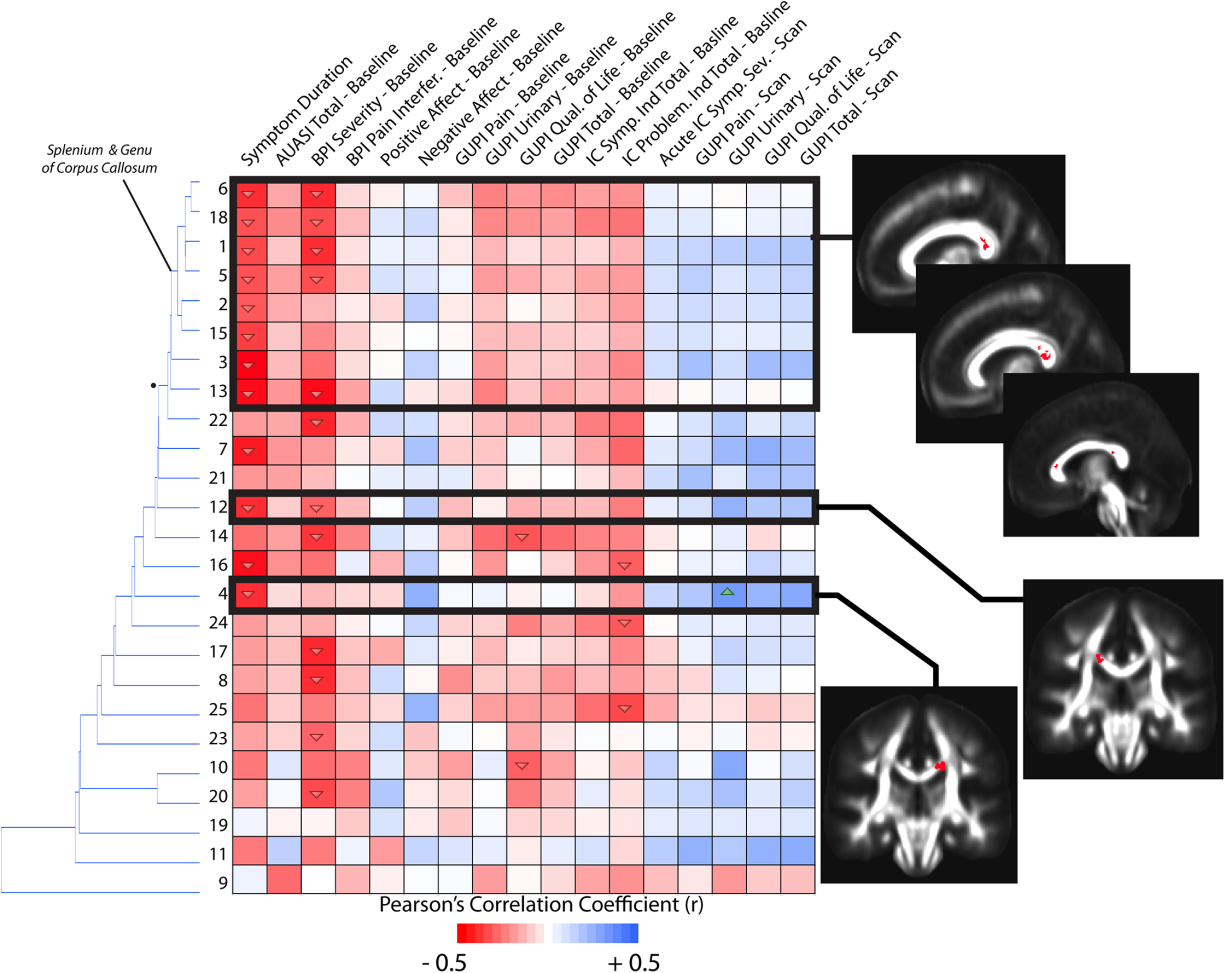
Correlation matrix between mean GA measurements and MAPP symptom scores in UCPPS patients, localized to ROIs identified as different between UCPPS and HCs on statistical parameter maps. Dendrograms on the left side of the correlation matrix show hierarchical clustering of ROIs based on the association between GA measurements and symptom scores. Images showing ROI localization are chosen for select associations. Up arrows within cells denote significantly positive correlations (*P<0*.*05*) and down arrows within cells denote significantly negative correlations (*P<0*.*05*). (Note the level of significance was not corrected for multiple comparisons in this exploratory analysis).

To test the hypothesis that the extent of specific microstructural changes within the brain may play a role in UCPPS symptoms, we explored the correlation between DTI and TDI measurements and various symptom scores in patients with UCPPS. The correlation matrix associating MD with symptom scores revealed interesting stratification of symptoms and anatomical regions (**Fig 8**). In general, longer symptom duration resulted in higher MD within regions observed to be significantly different between UCPPS and HCs. Additionally, the PANAS negative affect and scan scores had a tendency to show a negative association with mean MD. Hierarchical clustering revealed similar trends in association between MD and symptom scores in regions of the basal ganglia, internal capsule, coronal radiata typically projecting to sensorimotor regions, and areas of the genu of the corpus callosum and other prefrontal association fibers. Also, regions 6, 15, 5, and 7, which are all anatomical regions proximal to each other within the frontal lobe, showed additional associations with the IC problem index at baseline as well as symptom duration. Examination of the correlation matrix for FA revealed similar findings to MD, albeit with lower correlation coefficients (**Fig 9**). In particular, FA tended to decrease in subjects with longer symptom duration, while it increased in these regions with increasing scan scores and baseline PANAS negative score. Hierarchical clustering revealed similar associations between FA and symptom scores in regions of the genu and splenium corpus callosum, as well as the coronal radiata projecting to the sensorimotor regions, posterior thalamic projections, and regions in the internal capsule. Additionally, region 3, which was centered in the left sensorimotor integration areas, showed strong positive associations with negative affect at baseline and GUPI scan scores. Also, region 14, centered within thalamic regions thought to project to prefrontal regions, showed a strong negative association between FA and AUA total score at baseline as well as a strong negative association between FA and the total IC problem index at baseline. Dissimilar to FA and MD, few trends emerged from the correlation matrix for measures of track density and many regions did not appear to be strongly associated with particular groupings of symptoms (**Fig 10**). For correlations between mean GA in regions and symptoms (**Fig 11**), the strong trend for a negative correlation between symptom duration and decrease in GA in present, much like in the FA analysis. Most of the other trends (positive or negative correlations across regions) are the same for the GA and the FA analyses. One clinical symptom that was more strongly correlated with mean GA was BPI Severity, which while present in the FA analysis, showed a much stronger pattern in the GA analysis. The lack of apparent associations between track density and symptom scores may be partly due to only a single, large contiguous cluster included in the analysis (i.e. ROI 1). This cluster extends through the genu and splenium of the corpus callosum, bilateral coronal radiata, bilateral cingulate, bilateral superior and inferior longitudinal fasciculus. Together, these results suggest the degree of microstructural changes within specific areas of the brain is associated with symptom severity and symptom duration in patients with UCPPS.

## Discussion

Consistent with the hypothesis that UCPPS is characterized by microstructural changes within the brain, we observed significant widespread differences in regional DTI and TDI measurements in UCPPS patients when compared to HCs. Suggesting disease specificity of these changes for UCPPS independent of symptom severity, we also found differences between UCPPS and IBS patients in these parameters after correction for observed clinical and behavioral group differences. In general, patients with UCPPS showed elevated MD, lowered FA and GA, and lowered fiber track density compared with HCs in various white matter regions, consistent with decreased axonal or dendritic density, lowered directional coherence from axonal or dendritic projections, and a lower total connectivity, respectively. These changes were observed in specific regions of the brain involved in supraspinal sensory processing, including white matter tracts connecting the cingulate, temporal lobes, somatosensory integration areas, and frontal/prefrontal cortical projections. These findings are consistent with reported white matter changes in other chronic pain syndromes [36], including neuropathic pain [15] and IBS [10], and with a recent MAPP Network DTI study by Farmer *et al*.[37] showing widespread decreases in FA in similar anatomical regions in female UCPPS patients.

### White Matter Changes in Sensory Processing and Integration Areas

Although most of the brain demonstrated an increase in MD, lowered FA, and lowered fiber track density in UCPPS patients compared with HCs, we observed an *elevated* FA and decreased fiber track density in UCPPS patients in regions localized near known thalamic projections to prefrontal regions, regions in the basal ganglia and internal capsule, and somatosensory processing areas near known pelvic representation (**Fig 3**). This pattern of alterations appears paradoxical since an increased FA suggests increased directional coherence of axonal projections consistent with reinforced axonal or dendritic projections to these regions, while a decreased fiber track density suggests atrophy in those regions. It is conceivable that the increased FA within regions projecting to the pelvic somatosensory regions may result in strengthening of specific afferent projections to somatosensory regions as a consequence of constant painful stimuli. However, the reason for the observed decrease in overall track density, presumably reflecting atrophy of interconnections or altered representation of these body regions from reorganization, remains unclear. Even though the observed changes may reflect genetically/epigenetically determined brain changes which predispose an individual to the development of chronic pain, the observed correlations of these white matter changes with symptom duration provide more support for a causative relationship of increased viscerosensory input to the brain.

Our results support the hypothesis that significant microstructural reorganization occurs within regions of the basal ganglia, thalamus, and prefrontal regions in UCPPS patients, consistent with other chronic pain syndromes implicating altered cortico-basal ganglia-thalamus-cortical loops in pain modulation [38–40]. The current results demonstrating an increase in MD, *increased* FA, and decreased track density in these specific regions in patients with UCPPS suggests an imbalance of this loop as compared to HCs and may imply that patients with UCPPS have increased viscerosensory input as well as altered modulation of pain perception. This hypothesis is further supported by exploratory data in the current study showing similar associations between various DTI and TDI metrics in these particular regions and the correlation with various pain symptom scores, namely symptom duration and UCPPS severity. While deep gray matter regions represent a combination of gray matter and intersecting white matter fiber tracts, and thus complicates a solely microstructural interpretation of changes in these regions, the changes may reflect changes between gray and white matter that are nonetheless significant to these viscerosensory pathways.

Our results showing reduced FA throughout much of the brain and increased FA in specific areas appears associated with somatosensory function are consistent with several recent observations of cortical thickening and increased functional connectivity in somatosensory regions, associated with cortical thinning and decreased functional connectivity to the insula.[41] For example, compared to HCs, patients with ulcerative colitis, a chronic inflammatory condition of the large bowel, were found to have greater cortical thickness in the primary somatosensory cortex and in anterior cingulate cortical subregions, but lower cortical thickness in the insula.[42] Similarly, female IBS patients compared to HCs were found to have cortical thickening of primary somatosensory and motor regions, as well as significant cortical thinning of the insula. [43] Grey matter alterations in primary somatosensory cortex of UCPPS patients have also been identified in two recent MAPP publications, showing increased grey matter volume [44] and increased grey matter density [45] in UCPPS patients. A recent functional MRI study in female patients with interstitial cystitis/painful bladder syndrome showed increased functional connectivity and low frequency oscillation power within the primary sensory and motor cortices, and lower power within the posterior insula, compared with HCs. [46]

### Differences in White Matter Alterations Between UCPPS and IBS Patients

Although the current study observed microstructural changes consistent with a chronic pain signature similar to that found in other chronic pain conditions, we also identified differences between UCPPS and another, often overlapping visceral pain syndrome, e.g. IBS (**Fig 2**). We observed a significantly lower MD within the globus pallidus in the basal ganglia in patients with IBS compared with UCPPS patients, consistent with a recent study showing a similar reduced MD in IBS patients compared with HCs [10]. These results suggest a unique signature of changes within the globus pallidus in patients with IBS, which is not seen in UCPPS. Additionally, we observed a higher MD, lower FA, and lower track density within the corona radiata in UCPPS compared with IBS patients. Since white matter fibers within the corona radiate project to and from the cortex, along with project to the internal capsule, these results suggest a higher degree of microstructural changes in sensory, motor, and integration regions in patients with UCPPS. Unlike pelvic pain, the more widespread pain from IBS (referred from both small and large intestine) might be expected to have a different topographic representation on the somatosensory cortices.

### Sex Related Differences in White Matter Changes

We also observed differences in DTI and TDI measurements between male and female subjects unique to each patient group examined. Within the UCPPS group, few sex related differences in any DTI or TDI measurement were seen (though the differences were more pronounced in the GA analysis), while markedly different, almost opposite signatures were seen in HCs. Consistent with results from a previous study in IBS patients [10], male IBS subjects showed a higher FA and lower MD in the basal ganglia compared with female IBS patients, and this difference appeared to be exclusive to patients with IBS. Significant sex related differences in autonomic, perceptual, and emotional responses to visceral stimuli have previously been reported[47], some of which may be related to current findings and to previous sex related brain findings in IBS.[41] These findings suggest that the neuroplastic changes in the brain of UCPPS patients are similar between male and female patients, and that they may therefore be amenable to similar treatment strategies.

### Correlation of White Matter Changes with Symptoms

We found a decrease in FA and GA and increase in MD with increasing symptom duration, localized to areas of the anterior and posterior cingulum bundle, basal ganglia, frontal lobe white matter, thalamus, and somatosensory processing areas. A decrease in FA and GA and increase in MD within white matter areas is consistent with degenerative changes including demyelination [48–51] as well as decreased axon or dendritic density, caliber, and/or spacing [52–54]. The associations found between DTI changes in specific anatomical regions and symptom duration supports the hypothesis that the observed changes are not a primary brain abnormality predisposing to the disease, but may be secondary to chronically increased afferent input into sensory brain regions.

### Conclusions and Clinical Implications

Results from the current study may have important clinical implications, suggesting that long-term chronic pelvic pain results in measurable microstructural changes within specific areas of the brain involved in the processing and integration of sensory information from the pelvic area. These findings are most consistent with adaptation to chronically enhanced visceral afferent input to the brain. Such a causality is supported by the correlation of the observed white matter changes with symptom duration, but longitudinal studies are needed to determine the plasticity of the brain changes in patients who become asymptomatic either spontaneously or in response to specific therapies.

The observed white matter changes pose several important questions regarding the pathophysiology and the treatment of UCPPs patients. 1) Does the degree of white matter alterations predict which patient is likely to improve over time, or predict responsiveness to specific therapies? And are some of the changes more predictive than others? 2) If the observed brain changes are a consequence of chronically enhanced viscerosensory input to the brain, it indirectly implies that peripherally or spinally targeted therapies which reduce this viscerosensory input may be more effective than therapies targeted at the brain. 3) Which of the observed brain changes can be used as a specific biomarker of UCPPs as opposed to being a general biomarker for chronic pain? 4) What are the molecular and neuroanatomic changes underlying the observed white matter changes? Future studies using longitudinal assessments of subjects, interventional phenotyping studies and reverse translational studies in rodents are required to address these crucial questions.

## Supporting Information

S1 Fig The MAPP Research Network Study Group. (TIF)

S2 Fig Localized regions of significant site differences observed between UCLA and Northwestern University within UCPPS vs. HC comparisons for A) mean diffusivity (MD), B) fractional anisotropy (FA), C) fiber track density, and D) generalized anisotropy (GA). Significant clusters were determined by thresholding based on level of statistical significance (*P < 0*.*05*) and cluster-based corrections using random permutation analysis.
(TIF)
