# Supplementary figures and images for: Unique Microstructural Changes in the Brain Associated with Urological Chronic Pelvic Pain Syndrome (UCPPS) Revealed by Diffusion Tensor MRI, Super-Resolution Track Density Imaging, and Statistical Parameter Mapping: A MAPP Network Neuroimaging Study
Source: PLoS One. 2015 Oct 13;10(10):e0140250. doi: 10.1371/journal.pone.0140250 (PMC4604194; doi:10.1371/journal.pone.0140250)

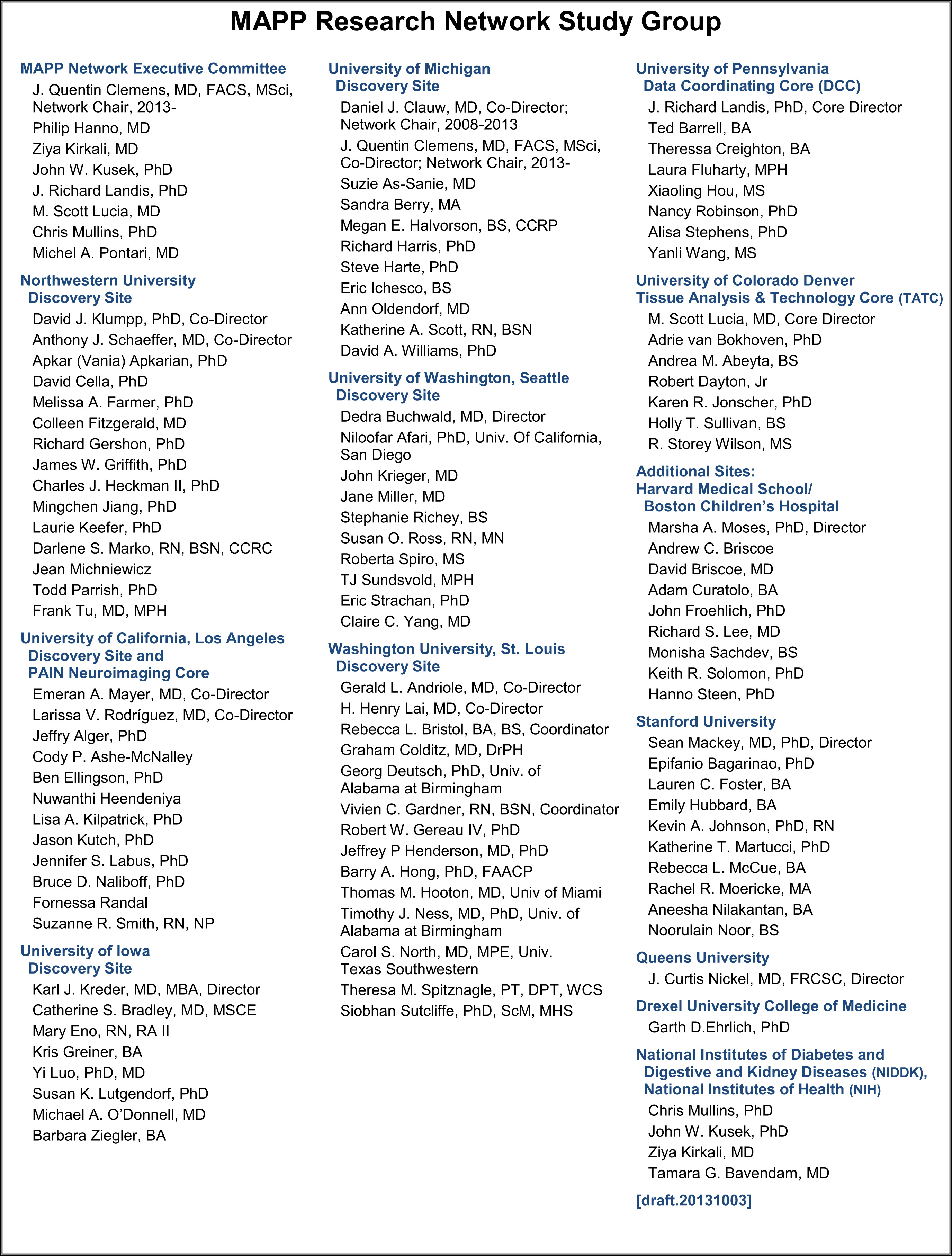

Supplement: S1 Fig — (TIF) [file pone.0140250.s001.tif]

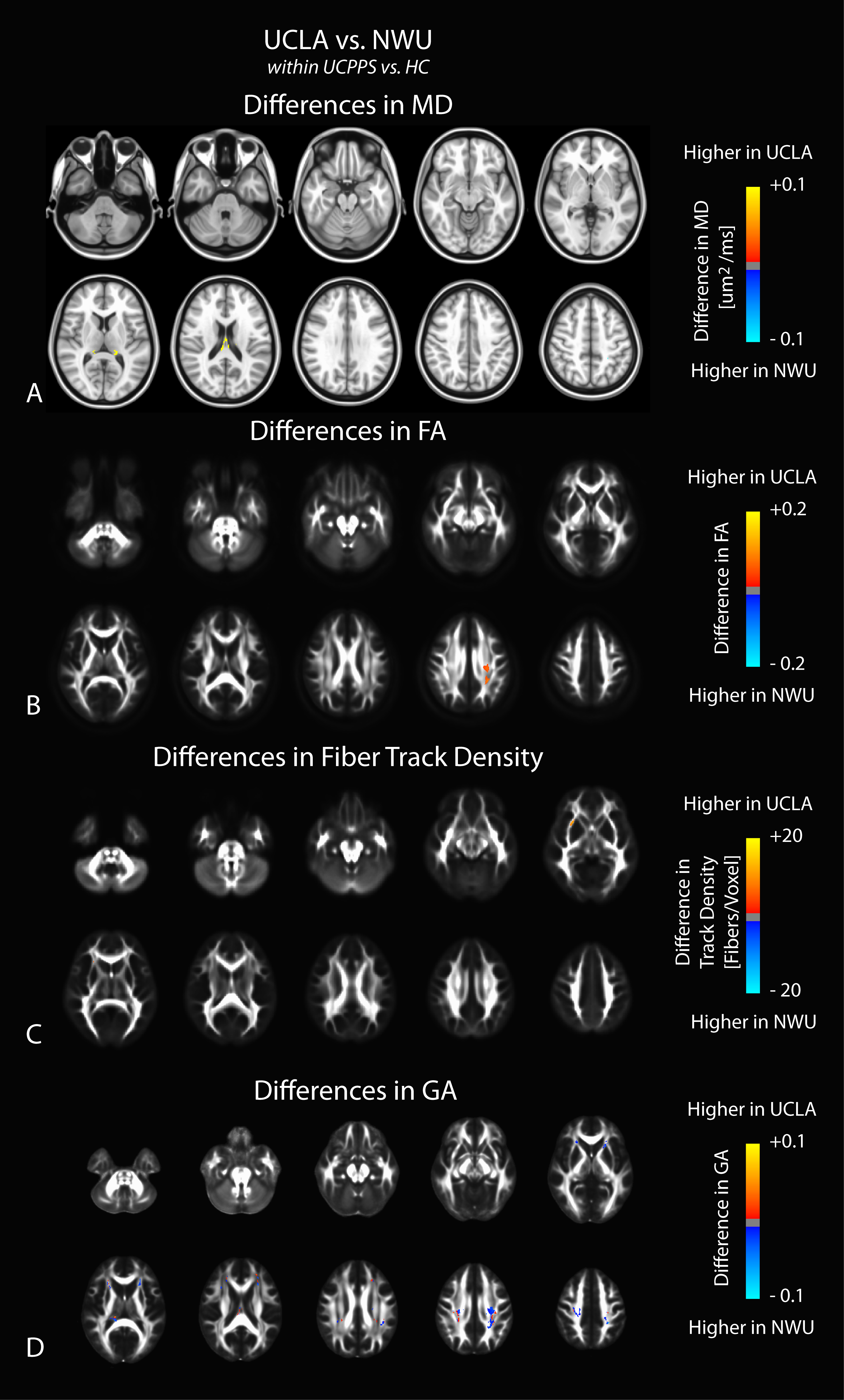

Supplement: S2 Fig — Significant clusters were determined by thresholding based on level of statistical significance (P < 0.05) and cluster-based corrections using random permutation analysis. (TIF) [file pone.0140250.s002.tif]
